# Optical properties of metamaterial split ring nematic colloids

**DOI:** 10.1038/s41598-019-43470-6

**Published:** 2019-05-07

**Authors:** Anja Pusovnik, Jure Aplinc, Miha Ravnik

**Affiliations:** 1 Faculty of Mathematics and Physics, University of Ljubljana, Jadranska 19, 1000 Ljubljana, Slovenia; 2 J. Stefan Institute, Jamova 39, 1000 Ljubljana, Slovenia

**Keywords:** Metamaterials, Liquid crystals, Liquid crystals

## Abstract

The fabrication of 3D bulk metamaterials, optical materials with sub-wavelength building blocks, is an open challenge, along with the tuning of their optical properties, such as transmissivity or exit polarization where a possible approach is to embed liquid crystalline materials into metamaterials and use their tunable birefringence. In this work, we explore using numerical modelling the photonic properties of a composite of split ring resonator colloidal particles, dispersed in nematic liquid crystal, which was optimised to enable self-assembly fully. Specifically, using generalised FDTD simulations for light propagation in birefringent profiles, we demonstrate the photonic response of single particles, 2D and 3D colloidal crystals. The material transmittance is shown to exhibit clear resonant behaviour with the resonances tunable with the birefringence in the order of ~5%. Electric and magnetic field modes emergent on the particles are shown, as affected by the surrounding nematic birefringence, both the in the slit region of the split ring resonator (SRR) particles as well as around the particles. Observed photonic response is further explained by introducing basic equivalent LC circuits. Finally, this work is aimed at developing soft and fluid metamaterials, which exhibit optical anisotropy in the photonic response as a potent mechanism for controlling the flow of light at wavelength and even sub-wavelength scales.

## Introduction

Metamaterials – artificially constructed composites of metal^1,2^ or dielectric^3,4^ inclusions much smaller than the wavelength of the incident light – enable novel ways for controlling the flow of light at microscale such as its polarization profile or wavelength-dependent transmission function^5^. The coupling between the metamaterials and light is highly conditioned by both complex shape of metamaterial elementary building blocks and their arrangement. By controlling the geometrical and material parameters of the metamaterial building blocks, different effects, such as negative refractive index^6^, complex vortex beams^7^, optical cloaking^8^ or lasing^9^ have been demonstrated. A particular example of the metamaterial building block is the split ring resonator (SRR) unit^10,11^, which was constructed in order to obtain a magnetic response from a material. Its form resembles a horseshoe: it is a loop with a slit-like separation. The round part acts as a magnetic element, i.e. as an effective turn of a coil, whereas the slit affects primarily the electric field, acting as a capacitor^12^. Such combined electric and magnetic response of the individual particle produces a resonant response of the material as a whole, which emerges in several ways, most notably as a change in the transmissivity spectrum^13^. Split ring resonator based metamaterials have been realized in experiments at different wavelengths from microwave^1^ to optical frequencies^6^.

One of the strong approaches for the fabrication of metamaterials is to use the self assembly or assembly of the colloidal metamaterial building blocks (particles) into an ordered structure, notably also through the use of liquid crystals, which by themselves can modify the flow of light^14–16^ and can be used for various optical applications^17,18^. Indeed, liquid crystalline colloids were rather recently shown to assemble into various stable colloidal configurations and structures^19,20^, from the configurations of spheres^21,22^ to different configurations of flat particles, such as square and circular platelets^23^. Optically and material-wise, liquid crystal colloids perform as birefringent fluids with spatially varying birefringence that have embedded dielectric or metallic particles of various sizes and/or shapes. The intrinsic elasticity of the liquid crystal creates effective forces of thermodynamic origin between particles, and causes the colloidal particles to assemble or orient in a specific way, forming stable clusters of particles, including particle chains^24^, 2D^25^ and 3D colloidal crystals^26^.

The merge of fields of optically birefringent liquid crystals and metamaterials has been studied mainly along two approaches^27–29^. Under first approach liquid crystal is used as as a host medium for the metallic or dielectric metamaterial colloidal particles, as for example in^30^, where the nanoparticles were immersed in and ordered by the disclination lines inside liquid crystal droplets. Under second approach, birefringent liquid crystalline materials are embedded as one of the components into the metamaterial composites, as in^31–33^, where liquid crystals were used as an addition to an existing metamaterial structure in order to obtain the desired properties of the composite. Notable interest lies in self-assembled liquid-crystalline metamaterials – i.e. colloidal particles that self-assemble into orientationally ordered liquids^34–36^, organize into larger clusters^37^ or position themselves along defect disclination lines^38^ – and in liquid crystals as material components, suitable for tuning, as the liquid crystals are susceptible to the influence of external electric fields^39^ and temperature^40^, or optical irradiation^41,42^. The influence of liquid crystals on metamaterial properties has also been shown in the field of metasurfaces, i.e. 2D metamaterials, where, as in 3D metamaterials, the advantages of liquid crystal tuning^43–45^ of the optical properties of the metasurface is explored, as well as using metasurfaces as an influence on liquid crystal, e.g. as a mask for liquid-crystal photopatterning^46^.

**Figure 1:**
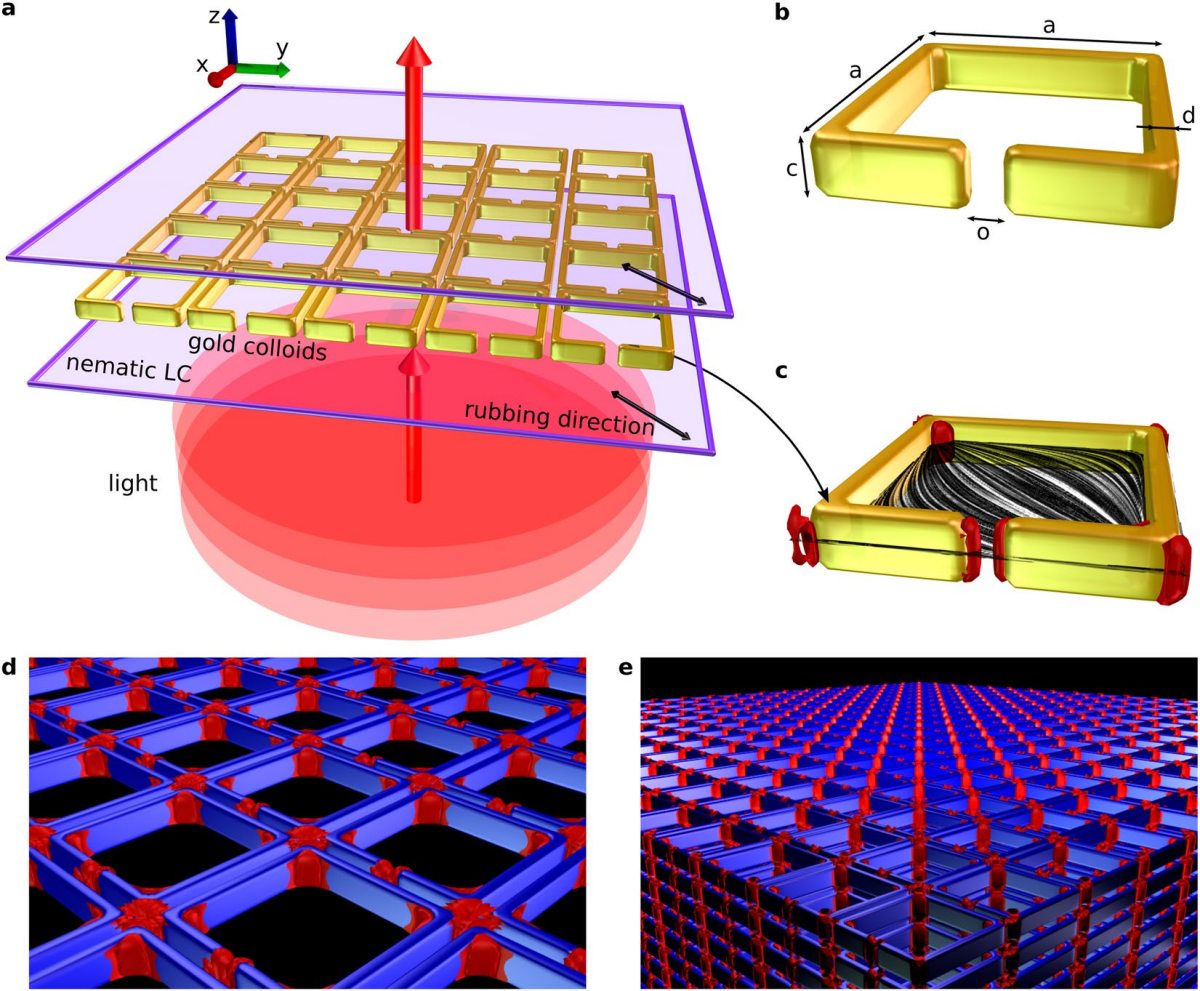
Self-assembled colloidal structures in nematic liquid crystals from split-ring resonator (SRR) particles. (**a**) Schematic depiction of 2D colloidal crystal made of gold SRR colloids within the nematic cell with planar anchoring on the surfaces. Red shows the wavefronts of the incident plane wave, which was in simulations polarized along *x* or *y* direction. Red arrow perpendicular to the disc represents the incident direction of light. (**b**) The geometrical parameters of SRR colloidal particles are: *a* = 500 nm, *c* = 100 nm, *d* = 50 nm, *o* = 30 nm. **(c**) Single colloidal SRR particle from within the 2D crystal with depiction of the director field (grey streamlines) and defects (red). (**d** and **e**) Simulated structures of 2D and 3D colloidal crystal of SRR particles, immersed in nematic liquid crystal. Particles are drawn in blue, red: defect structures of nematic liquid crystal.

In this paper, we present the analysis of the optical properties of golden split ring resonator particles assembled in nematic liquid crystal. The metamaterial cell and wavevector direction of light are sketched in Fig. 1(a), with SRR geometry elaborated further in Fig. 1(b) and the director field around the SRR shown in Fig. 1(c). We demonstrate the transmissivity response of one split-ring resonator particle, and 2D and 3D colloidal crystals of split ring particles (sketched in Fig. 1(d,e), respectively) in a nematic cell for two orthogonal polarizations of light, both in plane of the particle. We provide a qualitative explanation for the variations in the transmissivity function for each of the polarizations based on different modes of a LC-circuit. For 3D split-ring crystal, we determine the absorption of light transversing into metamaterial and approximate the absorption coefficient. We then explore the tunability of the optical properties of such 3D split ring nematic colloidal material with changing of the extraordinary refractive index of the liquid crystal, and show ~5% tuning of the resonant frequencies for the changes in birefringence of ~0.1. Finally, the tunable and self-assembling nature of these split-ring-liquid-crystal system is a step forward to the development of novel metamaterials, which are distinctly soft, fluid, possibly polimerisable and could allow for tuning with diverse stimuli including electric fields.

## Results

We show the emergence of resonant modes for light impinging on a single SRR particle embedded in a liquid crystal cell, and provide a qualitative explanation of the modes with the LC model. Then we simulate the light propagation through 2D and 3D photonic crystals of SRR particles in liquid crystals, and as an example of possible tuning of the nematic, we show the shifting of the transmissivity resonances for different values of extraordinary refractive index of the nematic.

### Photonic response of single SRR particle in nematic

**Figure 2:**
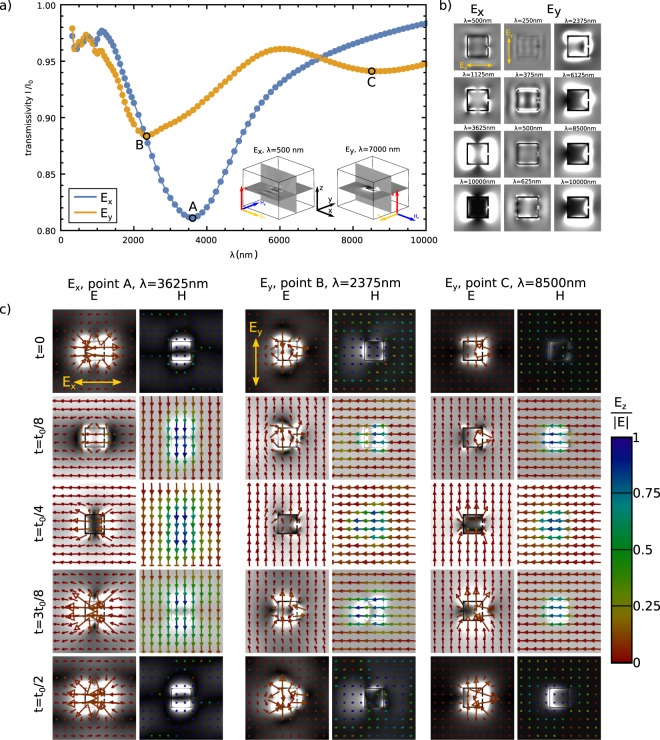
Photonic response of single SRR colloidal platelets in nematic. (**a**) The transmission spectra of a single colloidal metallic split-ring resonator, immersed in birefringent nematic, for two orthogonal (*y*- and *x*-) polarizations of incoming plane wave. The geometry of the structure and the directions of the incoming electric fields in the case of each polarization are shown in the insets, which also illuminate the response of the structure for two distinct regimes: diffractive regime for $\lambda \lesssim 500\,{\rm{nm}}$, and resonant metallic regime for $\lambda \gtrsim 1.5\,\mu m$. (**b**) Snapshots of the electric field amplitude, taken from the *xy*-plane cutting through the middle of the split-ring particle, and time-averaged across one wave period. (**c**) Snapshots of electric and magnetic field amplitudes (grayscale) at different times for the wavelengths of three resonances (minima) in the transmission diagram (points A, B and C in (**a**)). The times depicted are chosen as fractions of the wave period *t*_0_ for each individual wavelength, ranging from *t* = 0 to *t* = *t*_0_/2. The vectors show the *xy*-directions of the electric and magnetic field, whereas the color of the arrows indicates the relative amplitude of the out-of-plane (*z*) component compared to electric field amplitude |*E*|.

**Figure 3:**
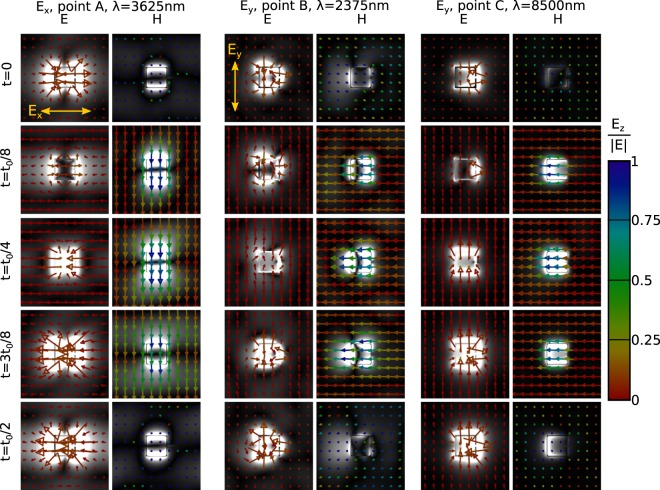
Resonant electric and magnetic field modes with subtracted background (light) field modulation. We plot $\sqrt{|\overrightarrow{E}-{\overrightarrow{E}}_{background}{|}^{2}}$, where ${\overrightarrow{E}}_{background}$ is the average value of points from all edges of the cell (i.e. effectively, the plane wave modulation), to be able to show and emphasize the actual field (modes) that emerge on the SRR particles. The $\overrightarrow{E}$-vector direction and size are the same as in Fig. 2(c). Note, the exchange of energy from magnetic into electric field and vice versa.

The photonic response of individual horse-shoe colloidal particles in nematic is shown in Figs 2 and 3. In Fig. 2(a), transmissivity for two orthogonal (*x*- and *y*-) polarizations is shown, with the polarization directions illustrated in the insets. The wavelengths at which the insets are shown are also chosen to emphasize the two distinctive regimes of the ratio of wavelength *λ* vs. size of the particle *a*. When the two are comparable ($\lambda \lesssim 500\,{\rm{nm}}$), diffraction on the particle is prevalent, whereas for $\lambda \gtrsim 1500\,{\rm{nm}}$, absorption on the particle is dominant. For *λ* ≤ 1500 nm, for both polarizations, different transmission peaks can be visible and can be connected to diffractive peaks and to dipole transmission resonances of higher order.

The change in the electric field and magnetic field upon transition from diffractive to absorptive regime is illustrated in Fig. 2(b), showing the electric field intensity in the plane of the split-ring nanoparticle for several wavelengths. For smaller wavelengths, we can see the wavelength of field oscillations around the nanoparticle is proportional to the incoming wavelength and is resonant when the ratio of particle length vs. incoming wavelength is a rational number. For larger wavelengths, the response of the nanoparticle is nearing the response of a point dipole; the higher the wavelength, more dipole-like response. The dipole resonances occur at approx. *λ* = 3625 nm and *λ* = 2375 nm for *x*- and *y*-polarization, respectively (points A and B in Fig. 2(a)). The difference between the resonances for *x*- and *y*-polarization can be attributed to the specific shape of the split-ring resonator particles: *x*-polarization experiences two (full) equal oscillating sides of the particle, whereas *y*-polarization one whole side but the other is split by the slit. Indeed, the resonance that emerges for the *y*-polarization at about *λ* = 8500 nm (point C in Fig. 2) is directly connected to the slit of the split-ring resonator. Its emergence is explained with the CR theory in the following subsection. For the *x*-transmissivity curve, since the slit does not influence the electric field oscillations, the corresponding resonance does not occur. The directions of electric and magnetic fields in the resonances are further demonstrated in Figs 2(c) and 3, where we plot time dependent snapshots of electric and magnetic fields. We see the emergence of *H*_*z*_ component (a component, perpendicular to the plane of the split-ring particle), which indicates a resonance in the magnetic field, and also and exchange of energy between electric to magnetic fields.

### Simple model of electromagnetic response of single SRR platelets in nematic

Metallic SRR colloidal particles perform as a small electromagnetic resonators or antennas due to their shape. In turn such response of the particles can affect the incident light, thus performing as metamaterials. The characteristics of particular SRR metamaterial depend on the geometry and dimensions of individual particles. Their photonic response can be understood and interpreted as the response of electromagnetic resonator that consists of two basic building blocks, namely an effective electric capacitor and an effective magnetic coil. The resonant frequency of electromagnetic resonator is defined as

1 $$\nu =\frac{1}{2\pi }\frac{1}{\sqrt{LC}},$$

where *L* is the inductance and *C* the capacitance. In the most elementary picture the inductivity of a coil (infinite coil) with number of turns *N* per coil length *l*_*L*_ and the surface area of the cross section *S*_*L*_ filled with material of permeability *μ* reads

2 $$L=\frac{\mu {\mu }_{0}{N}^{2}{S}_{L}}{{l}_{L}},$$

where *μ*_0_ is magnetic permeability of vacuum. The capacity of a plate capacitor with plate surface *S*_*C*_ and interplate distance *l*_*c*_ reads as

3 $$C=\frac{\varepsilon {\varepsilon }_{0}{S}_{C}}{{l}_{C}},$$

where *ε* represents the dielectric permittivity of the containing material and *ε*_0_ the permittivity of vacuum.

Of course, we are not using explicit coils or capacitors of such form; however, this concept can be used to provide rather simple and intuitive interpretation of our full calculated resonant behaviour of SRR nematic colloidal platelets.

**Figure 4:**
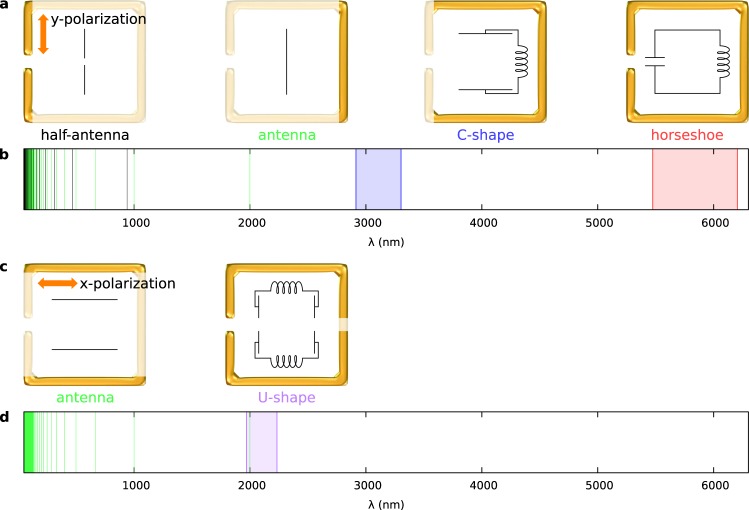
Basic excitation modes and the estimated absorption spectra of the metal SRR particles. (**a**) Excitation modes of SRR particle subjected to linear polarization along *y*-axis. (**b**) The estimated resonant frequency ranges of excitation modes presented in (**a**). (**c**) Excitation modes of SRR particle subjected to linear polarization along *x*-axis. (**d**) The estimated resonant frequency ranges of excitation modes presented in (**c**).

Effectively, different parts of the SRR particle excite and take the role of capacitor or a coil at distinct frequency regimes and polarizations. When the incident light is polarised in the direction of *y*-axis, there exist several possible excitation modes (Fig. 4(a)) with distinct resonant wavelengths: (i) Horseshoe, where the slit performs as a capacitor and the whole frame of the split-ring resonator particle acts as the coil, (ii) C-shape, where the parallel arms of the SRR particle perform as a capacitor and half of the frame acts as the coil, (iii) Antenna modes, where the side of the SRR particle opposite to the slit acts as an antenna, and (iv) Half-antenna modes, where the side of the SRR particle with the slit acts as two smaller antennas. When the incident light is polarized in the direction of *x*-axis, two excitation modes are expected (Fig. 4(c)): (i) U-shape with another stationary point of $\overrightarrow{E}$ opposite the slit, which effectively gives two capacitors and two coils with equal *L* and *C*, and (ii) Antenna modes along the two horizontal edges of the SRR particle.

The dielectric permittivity (tensor) in the slit or in the interior of the SRR particle is spatially dependent, with full variability shown in Fig. 1(c). We consider only the two limiting regimes whether the director is parallel or perpendicular to the electric field in the slit. The corresponding limiting effective permittivities are ${\varepsilon }_{\parallel }^{{\rm{eff}}}=\bar{\varepsilon }+{\varepsilon }_{a}^{{\rm{mol}}}\frac{2S}{3}$ and ${\varepsilon }_{\perp }^{{\rm{eff}}}=\bar{\varepsilon }-{\varepsilon }_{a}^{{\rm{mol}}}\frac{S}{3}$, which can be used to estimate the minimum and maximum frequencies of the resonances. For 5CB – as an example of a typical nematic material – and nematic degree of order *S* = 0.533 the difference between ${\varepsilon }_{\parallel }^{{\rm{eff}}}$ and ${\varepsilon }_{\perp }^{{\rm{eff}}}$ is around 13%, but can be tuned up to 25% if increasing *S*. The wavelengths of these effective estimates of the resonances of each excitation mode, calculated using actual particle dimensions, are presented in Fig. 4. The wavelengths of the considered resonances reside in the infrared part of the spectra, whereas resonant wavelengths depend linearly on the particle size and may be changed by resizing the particles. Indeed, the calculated estimates of resonant frequencies (Fig. 4) are in good qualitative agreement with the results of full numerical calculations (Fig. 2), providing a qualitative interpretation of the observed transmittance spectra.

### Photonic response of 2D and 3D nematic SRR colloidal crystals

**Figure 5:**
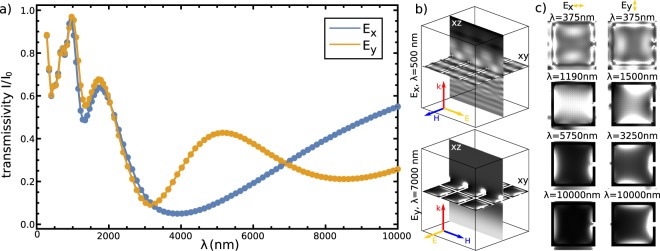
Photonic response of 2D nematic colloidal crystal of SRR particles. (**a**) Transmission spectra of a 2D crystal of split-ring resonator particles for E-field polarized along the slit (*x*-polarization) and across the slit (*y*-polarization). (**b**) The geometry of the calculated structure for *x*- and *y*-polarizations, shown for *x*- polarization in the diffractive regime (*λ* = 500 nm) and for *y*-polarization in the resonant regime (*λ* = 7000 nm). (**c**) Snapshots of averaged electric field, taken from the xy-plane running through the split-ring particles. Note the effective transition from the diffractive regime towards a dipole-like oscillations of electric field with increasing wavelengths.

Figure 5(a) shows transmissivity for light passing (from below) through the 2D colloidal crystal; Fig. 5(b,c) show selected electric field profiles at distinct (resonant) frequencies. Clearly, the transmittance of the 2D crystal reveals also distinct resonances for both *x*- and *y*- polarised incident light. The minima in the transmittance that correspond to resonances are strong – of multiple 10% – and show profound response over a notable frequency range of the material (for example, compare to order of magnitude ~1% effects with single particles). With increasing wavelength, the field distribution is nearing to be dipole-like for both polarizations. The non-metallic resonances of the 2D-material ($\lambda \lesssim 1500\,{\rm{nm}}$) show greater variations in amplitude with respect to the one-particle transmissivity function. For example, at *λ* = 875 nm, both polarizations exhibit a maximum transmissivity which is nearly equal to 1, whereas the minima drop significantly lower because of the higher density of the split-ring particles. Similarly, for $\lambda \gtrsim 1500\,{\rm{nm}}$, absorption of gold is considerable, so in 2D and 3D crystals again the larger packing fraction of golden particles (i.e. effective occupied volume) leads to stronger absorption, as compared to Fig. 2(a).

Interestingly because of the cross-talk between the neighbouring particles, the ‘slit resonance’ for *y*-polarization shifts to slightly larger wavelengths, i.e. from *λ* = 7875 nm for one particle to *λ* = 8625 nm for the two-dimensional array of split-rings.

**Figure 6:**
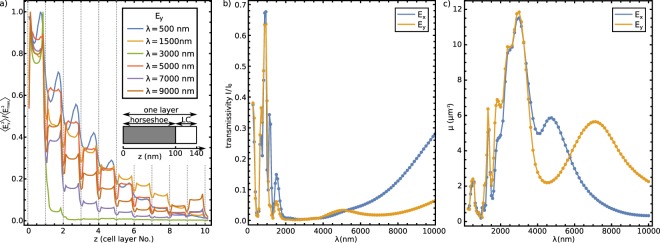
Photonic properties of 3D split-ring crystal: 10 layers  of particles. (**a**) Time-averaged $\langle {\overrightarrow{E}}^{2}\rangle$ for incoming *y*-polarization, normalized with the maximum field $\langle {\overrightarrow{E}}_{\max }^{2}\rangle$ for different wavelengths. The average *xy*-values are plotted along the *z* axis in units of cell layer. (**b**) Transmissivity of such a 3D crystal for different wavelengths of incident light. (**c**) Estimated absorption coefficient for both ${\overrightarrow{E}}_{x}$ and ${\overrightarrow{E}}_{y}$, and different wavelengths of incident light, using $\langle {\overrightarrow{E}}^{2}\rangle =A\,\exp (\,-\,\mu z)$.

Photonic response of 3D SRR colloidal crystal is shown in Fig. 6. In Fig. 6(a), we plot the effective electric field intensity ${\overrightarrow{E}}^{2}$, averaged over time and across the *xy*-plane, inside the 3D crystal for several vacuum wavelengths. The values are normalized with respect to the maximum value of the electric field inside the crystal for each specific incoming wavelength. We see a gradual effective decline of averaged electric field when propagating through the particle layers of the crystal, which is specific for individual wavelengths. Note, that we clearly observe a notable drop in the averaged electric field in the narrow regions between the sub-sequent layers of particles, which is a consequence of effectively higher refractive index of the liquid crystal layer between the metallic particles, as compared to effective refractive index of particles. Transmissivity for light passing ten layers of particles is plotted in Fig. 6(b). The resonances are even more pronounced than in the 2D materials, and the transmissivity falls almost to 0 for large wavelengths. To analyze the decline in averaged electric field in Fig. 6(a), the curve $\langle {\overrightarrow{E}}^{2}\rangle =A\,\exp (\,-\,\mu z)$ was fitted to the data and the effective absorption coefficient *μ* was extracted (Fig. 6(c)). The frequency variation of the absorption coefficient is strongly related to the variation of transmissivity, but shows finer details at the wavelengths where absorption is high, which is roughly at wavelengths between approximately 2000 and 8000 nm. A good agreement between the minima in transmissivity and maxima in absorption are found, with better than or cca ~5% correspondence.

### Tunability of nematic split-ring resonator metamaterials

**Figure 7:**
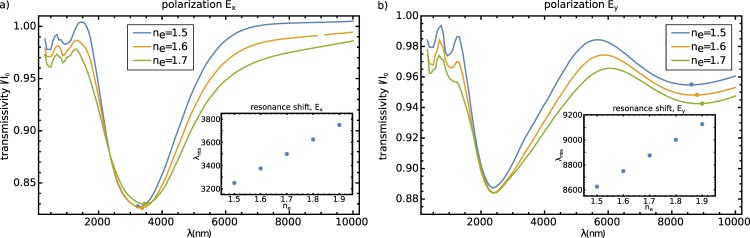
Tuning of the metamaterial resonances with varying extraordinary refractive index *n*_*e*_. Transmissivity functions for (**a**) *x*-polarized, (**b**) *y*-polarized incoming electromagnetic wave for *n*_*e*_ = 1.5, 1.6 and 1.7. Insets: shift of the minima in the transmissivity diagram with changing *n*_*e*_.

Liquid crystals are an interesting material especially in terms of tunability with the external electric field and/or temperature. By raising the temperature, the degree of order and the optical anisotropy drops. The optical anisotropy, in turn, influences the optical properties of the split-ring structure, notably including the exact positions of the transmittance resonances. In Fig. 7, as an example of tunability, we show the tuning of the transmissivity spectra by changing the value of the extraordinary refractive index *n*_*e*_ (roughly, the birefringence), for *E*_*x*_ and *E*_*y*_ polarized light. The tunability emerges both in the exact values of the transmissivity as well as the exact values of the resonant frequencies. For *E*_*x*_ polarization, the positions of the resonance is shifting for 300 nm towards higher wavelengths when Δ*n*_*e*_ = 0.2, which is ≈8% of the value for the isotropic medium. For *E*_*y*_ polarization, the wavelength shift is strongest for the ‘slit resonance’ at about *λ* = 8700 nm and is for Δ*n*_*e*_ = 0.2 equal to ≈3% of the value for isotropic medium, i.e. Δ*λ* = 350 nm. The change in the resonant frequency is the result of the change in the effective birefringence within the slit region: the optical axis within the slit is oriented approximately at an angle of 45° to the slit; therefore, any change in the *n*_*o*_ and *n*_*e*_ produces a change in the effective dielectric capacity of the slit which changes the resonant frequency of such a LC circuit.

## Discussion

More generally, developing metamaterials that rely on (colloidal) self assembly has – in principle – no limitation on the type of material of the particles, meaning that metallic, dielectric, or other type of material particles can be used. Actually, today, a broad range of colloidal particle types, shapes and materials are studied and developed, that could be of potential interest for developing soft or liquid metamaterials^31,47–50^. In addition to metallic, also of specific interest for metamaterials would be all-dielectric materials that utilize different methods to achieve the desired resonant response, for instance Mie or Fano resonances, and for which the split-ring resonator shape of the constituent particle used in this work is not appropriate; most typically spherical or cubic particles are employed^3^. The particles (and dispersions) are created with different chemical and physical process, including with high yield and atomic precision^51,52^. Specifically, in optically birefringent liquid crystal colloids, particles and structures of diverse functunalities, sizes and shapes were developed for metamaterials, including 2D and 3D colloidal crystals^53^ and even quasicrystals^54^ and particle knots^55^.

In the article, we demonstrate the performance of nematic colloidal SRR colloidal particles as optical metamaterials that exhibit multiple resonances that can be controlled and tuned by the colloidal particle structure and the material birefringent properties. Firstly, we analyze the response of one split-ring resonator embedded in a (planar) nematic cell, where we observe a spectrally dependent transmissivity response with pronounced minima in the transmissivity function. These minima were, with a LC circuit analogy, shown to correspond to the resonances of electromagnetic fields in the split-ring resonator geometry. We have analyzed the transmission function of 2D and 3D colloidal crystals from SRR particles, revealing a qualitatively similar transmissivity function as for the single split-ring, but different for the effects of interparticle field coupling that is revealed as an effective shift of the resonances towards higher wavelengths compared with the one-particle transmissivity function. We have explored tuning of such nematic metamaterials by varying the specific resonances of one split-ring particle with changing of the extraordinary refractive index of the liquid crystal. The wavelength-shifting of the resonances, was shown to be of the order of 8% for resonance at 3225 nm and *E*_*x*_ polarization and on the order of 3% for the slit-dependent resonance of *E*_*y*_ polarization at 8625 nm. This work is a contribution towards optically controlled, tunable and self-assembled metamaterial systems, where specifically we are interested in using and developing the anisotropic response of the metamaterial.

## Methods

The study of split-ring resonator particles in nematic was developed within two main objectives. Under first study, we explored the actual assembly of split-ring particles in nematic background, where the key challenge was to optimize the SRR particle shape to avoid formation of metastable structures. This work is in short presented below, but more extensively in separate publication^56^. Whereas the second part of the study really focused on the photonic response of such materials – and the results are presented in this paper.

### Optimization of colloidal shape

The design of colloidal structures from SRR particles in nematic was made with the Landau-de Gennes (LdG) free energy approach^57^ and is explained in more detail in our connected article^56^, where various split-ring-resonator-like colloidal geometries along with their orientation in the liquid crystal cell were studied for the possibility of self-assembly. The total free energy *F* for each of the studied geometries was minimized numerically by using an explicit Euler relaxation finite difference scheme on a cubic mesh. As the result of optimization, we obtain the split-ring resonator particle with the dimensions as indicated in Fig. 1, which in liquid crystal orients roughly at a 45° angle between the symmetry axis of the particle and the imposed liquid crystal director, and more importantly does not indicate pair interaction potential that would lead to multiple (irregular) metastable structures, that are otherwise frequently observed in nematic colloids. When introducing multiple particles the interactions led to the self-assembly of these platelet-like colloids into 2D and 3D crystals.

### Simulations of light propagation

The simulations of light transmission were performed with the finite-difference time-domain (FDTD) approach^58^, custom developed in our group to directly account for full spatial variability of the birefringence. FDTD consists of leapfrog stepping in time for electric and magnetic field $\overrightarrow{E}$ and $\overrightarrow{H}$ on a staggered cubic mesh. The standard Yee grid of the FDTD code was adapted for light propagation through liquid crystals: each of the $\overrightarrow{E}$ and $\overrightarrow{H}$ components is calculated in every point of the array, which reduces the memory efficiency of the calculation, but improves the stability of the code, allowing for the calculation of light propagation through a structure with arbitrary birefringence.

The birefringent profile around SRR colloidal particles directly affects the transmission of light via the effective permeability and permittivity of the surrounding medium, which is in our case nematic liquid crystal. Permittivity *μ* of a typical nematic is very close to one, but differently permeability *ε* exhibits anisotropic behaviour. Dielectric tensor *ε* that characterizes nematic birefringence and is generally strongly spatially dependent is closely related to the tensor order parameter *Q*_*ij*_ via

4 $$\varepsilon =\bar{\varepsilon }{\bf{1}}+\frac{2}{3}{\varepsilon }_{a}^{{\rm{mol}}}{\bf{Q}},$$

where **1** is unit tensor, $\bar{\varepsilon }=\frac{1}{3}(2{\varepsilon }_{\perp }+{\varepsilon }_{\parallel })$ is the isotropic part of *ε* and ${\varepsilon }_{a}^{{\rm{mol}}}={\varepsilon }_{\parallel }^{{\rm{mol}}}-{\varepsilon }_{\perp }^{{\rm{mol}}}$ is the anisotropic part of the molecular electric permittivity - the permittivity of nematic with all molecules perfectly aligned. The molecular anisotropy ${\varepsilon }_{a}^{{\rm{mol}}}$ needs to be multiplied with the degree of order *S* to yield macroscopic material anisotropy *ε*_*a*_. The birefringent profile was determined with the free energy minimization as explained in previous section. If not indicated differently, the nematic birefringence was taken to be Δn = 0.2, with ordinary refractive index n_o_ = 1.5 and extraordinary refractive index n_e_ = 1.7. For the metallic particle, the real and imaginary parts of frequency dispersion for gold was implemented via the auxiliary differential equation (ADE) algorithm: with two additional equations for two auxiliary variables we represent the frequency-dependent Drude-Lorentz model of permittivity^59^, with data for gold taken from^60^. Since in the visible and IR range the dispersion of the liquid crystals is small in comparison with the dispersion of Au^61–63^, it was not taken into account. Note that the frequency dispersion of Au in the regime of our calculations changes from a low-absorption material with real part of the refractive index *n* > 1 (i.e. a dielectric) to a high-absorption material with real part of the refractive index *n* < 1 (i.e. a metal).

The incident light was assumed to be a plane wave source, entering the cell from the bottom, and propagating generally along *z* (see Fig. 1). In all of the cases, periodic boundary conditions were assumed along the *x*- and *y*-sides of the simulation cell. The top and bottom (along *z*-axis) of the calculation cell were enveloped with a boundary layer and a layer of PML^64^ to ensure the outgoing wave would exit the calculation cell without reflection at the upper edge of the cell.

The cell used for the calculation of the photonic response of single SRR particles in nematic was 200 × 200 × 500 voxels with resolution of 10 nm/vox, which gives the physical size of the cell to be 2 × 2 × 5 μm. The dimensions of the golden split-cell platelet were, as indicated in the general scheme of Fig. 1, 500 × 500 × 100 nm with the arm thickness of 50 nm and the gap size of 30 nm. The split-ring particle is centered in the middle of the calculation cell and surrounded with the nematic profile which was obtained from the free energy minimization calculations. The director field is mostly oriented at about 45° with respect to *xy*-plane, with the exemption of the director field close to the particle, where, because of the interaction with the particle surfaces that impose planar anchoring, the director field (optical axis) is aligned parallel to the particle. We have simulated the propagation of light for a wide range of vacuum wavelengths *λ*, ranging from 250 nm to 10 μm. The smallest wavelengths are comparable to the typical particle size (500 nm), which gives rise to diffractive effects around the particle, whereas for larger wavelengths, primarily absorptive effects determine the response of the structure. Because of periodic boundary conditions, the calculations show some interference for wavelengths of about the same size. However, they do not affect the specific shape of the transmissivity function, since diffraction does not change the amount of energy flowing through the structure. For the calculations of the two-dimensional photonic crystal, the cell used was 54 × 54 × 500 voxels in size with resolution of 10 nm/vox, which gives the physical size of the cell to be 540 nm × 540 nm × 5 μm. The platelets were of the same size as in the previous example (500 × 500 × 100 nm), placed in the centre of the cell. The cell was bounded with periodic boundary conditions in the *x*- and *y*-direction. The director field of the structure was again obtained with free-energy-minimization calculations. With 3D photonic crystal, the *xy*-size of the cell was the same (540 × 540 nm), while the height of the cell for the single particle was 140 nm, which accounts for the *z*-distance of 40 nm between stacked colloidal particles of size 500 × 500 × 100 nm. Ten of the cells were stacked on top of each other, periodic boundary conditions were assumed in the *x*- and *y* directions together with a plane wave source. For both 2D and 3D systems, we have simulated the propagation of light for vacuum wavelengths *λ* ranging from 250 nm to 10 μm.
